# Network Neuromodulation of Opioid and GABAergic Receptors Following a Combination of “Juvenile” and “Adult Stress” in Rats

**DOI:** 10.3390/ijms21155422

**Published:** 2020-07-30

**Authors:** Omer Horovitz, Ziv Ardi, Shiri Karni Ashkenazi, Gilad Ritov, Rachel Anunu, Gal Richter-Levin

**Affiliations:** 1 Psychology Department, Tel-Hai Academic College, Haifa 1220800, Israel; horovitz.omer@gmail.com; 2 Department of Behavioral Sciences, Kinneret Academic College on the Sea of Galilee, Sea of Galilee 15132, Israel; 3 Sagol Department of Neurobiology, University of Haifa, Mount Carmel 31095, Israel; shirik.ashkenazi@gmail.com (S.K.A.); glevin@univ.haifa.ac.il (G.R.-L.); 4 The Institute for the Study of Affective Neuroscience (ISAN), Mount Carmel 31095, Israel; giladritov@gmail.com (G.R.); anunu@univ.haifa.ac.il (R.A.); 5 Psychology Department, University of Haifa, Mount Carmel 31095, Israel

**Keywords:** juvenile stress, PTSD, GABA, opioid, neuromodulation, animal models

## Abstract

Early life stress is suggested to alter behavioral responses during stressful challenges in adulthood and to exacerbate pathological symptoms that reminisce posttraumatic stress disorder (PTSD). These effects are often associated with changes in γ-Aminobutyric acid type A (GABAA) and κ opioid receptor expression and neuromodulation of the limbic system. Anxiety-like and stress coping behaviors were assessed in rats exposed to stress in adulthood on the background of previous exposure to stress in juvenility. Two weeks following behavioral assessment in adulthood, GABAAR α1 and α2 subunits and κ opioid receptor expression levels were measured in the medial prefrontal cortex (mPFC), nucleus accumbens (NAc), amygdala, and periaqueductal gray (PAG). To illustrate changes at the network level, an integrated expression profile was constructed. We found that exposure to juvenile stress affected rats’ behavior during adult stress. The combination of juvenile and adult stress significantly affected rats’ long term anxious-like behavior. Probabilities predicting model integrating the expression of GABAA α1-α2 and κ opioid receptors in different brain regions yielded highly successful classification rates. This study emphasizes the ability of exposure to stress in juvenility to exacerbate the impact of coping with stress in adulthood. Moreover, the use of integrated receptor expression network profiling was found to effectively characterize the discussed affective styles and their behavioral manifestations.

## 1. Introduction

Posttraumatic stress disorder (PTSD) is a common and disabling disorder that occurs after an exposure to a potentially life-threatening traumatic event. Unlike other psychiatric disorders, PTSD diagnosis requires the occurrence of a specific type of traumatic event to precede the appearance of the clinical syndrome [1]. However, while the occurrence of traumatic events is unfortunately quite common, PTSD develops in only about 10–20% of exposed individuals, indicating the role of pre-trauma risk factors in the development of the disorder [2,3]. Exposure to stress early in life was proposed as one of these risk factors [4,5]. In line with that, it was previously indicated that individuals who experience early life stress were more likely to develop PTSD in adulthood compared to individuals with no such history [6,7].

Recent years have witnessed a growing interest in the development of effective and valid animal models for the long-term consequences of exposure to stress early in life (ELS). It was previously shown by others and us that an exposure to acute stress in juvenility—a period that is suggested to be of relevance to human childhood [8]—can increase vulnerability to stressful events in adulthood [9,10]. This exposure was associated with an impaired ability of animals to cope with stressful challenges in adulthood, lasting alterations in GABA-ergic functioning and with alterations in levels of circulating corticosterone (for a review, see [11]).

Two of the most recurrent findings relating to the neurobiology of PTSD are decreased activation of medial prefrontal cortex (mPFC) and increased amygdala activation [12,13]. It was proposed that hyperactivation of the amygdala might explain the failure of extinction of enhanced responses to fearful stimuli, a key component assumed to contribute to reexperience in PTSD. In addition, activation of the mPFC—an assumed regulator of amygdala activation—was found to be decreased during fearful memory retrieval in PTSD patients [14], an effect that could further exacerbate PTSD symptoms. Since the behavioral and neuronal system manifestations of PTSD bear strong resemblance to behavioral and neural alterations observed in fear-conditioned rodents, it was hypothesized that PTSD involves enhanced fear conditioning-related molecular, cellular, and anatomical mechanisms [14,15,16]. In accordance, measurements of activation by immediate early gene expression following retrieval of fear memory in rats found increased activation of the central nuclei (CeA) and basolateral region (BLA) of the amygdala [17]. In addition, activation of the prelimbic division (PRL) and infralimbic division (IL) of the mPFC changes following extinction of fear memory [18].

However, the stress response involves the activation of many brain structures [19,20]. A limbic network which consists mainly of the hippocampus, amygdala, and prefrontal cortex is a key contributor to the behavioral responses to stress [21]. Still, other brain regions such as the periaqueductal gray (PAG) have long been implicated in stress-related behaviors [22,23]. Interactions of the mPFC and amygdala with “bottom-up” regulation mechanism attributed to the PAG were recently indicated [24]. For example, Cole and McNally (2008) found that both the BLA and ventral periaqueductal gray (vPAG) activation were necessary for fear learning [25]. Moreover, neither of these regions alone was sufficient for fear learning to occur, suggesting that the amygdala and PAG make dissociable but complementary contributions to fear conditioning. The nucleus accumbens (NAc), a brain region mainly attributed to positive behavioral tendencies [26], has also been implicated in the response to stress by regulating GABAergic control over the hypothalamic–pituitary–adrenal HPA axis [27]. The NAc also receives many projections from the mPFC [28,29] and BLA [30]. In accordance with that, increased expression of the immediate early gene zif268 in both the core and shell subregions of the NAc was documented following the retrieval of contextual fear memory [31]. 

Taken together, a network involving the mPFC, amygdala, NAc, and PAG might play a role in the encoding, formation, and extinction of fearful memory. When dealing with individuals exposed to childhood trauma, it is hypothesized that they will exhibit a network disturbance in their emotional “top-down” and “bottom-up” regulatory systems, leading to maladjusted behavior during and after adult stress [32,33]. Understanding the effects of exposure at the network level may be of importance for the understanding of PTSD and its neurobiological manifestations.

Abnormal neuromodulation of γ-aminobutyric acid (GABA) was suggested to be involved in the pathophysiology of mood disorders [34,35]. In particular, γ-Aminobutyric acid type A (GABAA) receptor (GABAAR) was implicated in anxiety [36]. GABAAR is comprised of different subunits, with emphasis on α subunits, which contribute to its functional characterization [37,38,39,40]. Indeed, specific alterations in the expression of α1 and α2 subunits in the BLA of rats following exposure to juvenile and subsequent adult stress was reported [41].

The κ-opioid receptors (KORs) were also suggested to be involved in depressive and anxiety disorders [42,43], and the opioid system is regulated by an exposure to early life stressors [44,45]. The activation of KOR was shown to produce dysphoric and pro-depressive-like effects in both animals and humans [46]. Increased KOR signaling in limbic brain regions such as the NAc was suggested to mediate depressive-like behaviors following an exposure to stress [47]. Moreover, it was shown that administration of KOR antagonists produces a unique combination of antidepressant and anxiolytic-like effects in rats [48], suggesting that this class of drugs might be effective for the treatment of depressive and anxiety disorders.

We examined here alterations of expressions of GABAAR and KOR in a stress-related network (i.e., the mPFC, NAc, amygdala, and PAG) in adult stressed rats on the background of previous exposure to stress in juvenility. The “juvenile stress” paradigm employed emphasizes several factors of acute stress like novelty, restraint, uncontrollability, and unpredictability [49]. To evaluate the impact of “juvenile stress” in adulthood, animals were tested on the Elevated Plus Maze (EPM) test and then were exposed to the form of the Two-Way Shuttle Avoidance (TWSA) task, both as a test for the impact of “juvenile stress” and as an “adult stress” protocol. Since it was previously suggested that subjecting juvenile stressed animals to an additional stressful challenge in adulthood resulted in heightened anxiety levels [9,49], animals were further examined in the Elevated Zero Maze (EZM) and Social Interaction test (SI) 3 days following exposure to the TWSA task. Finally, GABAAR α1 and α2 subunits as well as KOR expression levels were assessed in the mPFC, NAc, amygdala, and PAG two weeks following the last behavioral assessment.

## 2. Results

### 2.1. The Exposure to Juvenile Stress by Itself Has Limited Effect on Behavior in Adulthood

**Figure 1:**
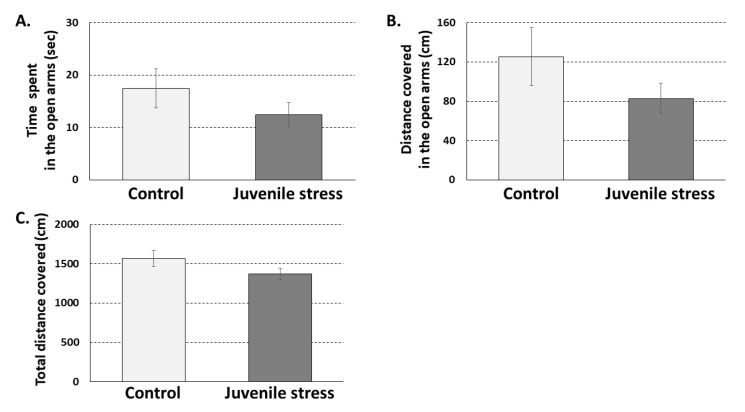
Rat behavior in the elevated plus maze (EPM) 28 days following exposure to juvenile stress: No significant differences were observed between control (*n* = 28) and juvenile stress exposed rats (*n* = 28) in the amount of time spent (**A**) and in activity levels (**B**) in the open arms of the EPM. In addition, no significant difference was observed between the groups in total activity levels in the EPM arena (**C**).

Elevated plus maze test (EPM) was conducted 28 days after exposure to the juvenile stress and three days before exposure to the adult stress. Previous work by our group tested the effects at different time points of the exposure to stress at juvenility. We mainly focused on 60 postnatal day PND because rats reach their sexual maturity at this age and are considered young adults [11]. One month following exposure to juvenile stress, profound effects of the stress exposure were observed, mainly on the ability to cope with a stressor in adulthood [9,10,11]. It is worth noticing that we also observed significant effects of the exposure to juvenile stress at later periods (e.g., 2–3 months following exposure [10]). However, these effects were not markedly different from those observed one month following exposure to stress in juvenility (for a comperhensive review of the topic, please see [11]). Specifically, no significant effects were found for juvenile stress alone on anxiety-like behaviors in the EPM (Figure 1) (time spent in open arms, distance traveled open arms, and total distance covered (t(54) = 1.145, n.s; t(54) = 1.271, n.s; and t(54) = 1.578, n.s. respectively)). The rather low level of basal exploration (10–20 s or approximately 5% of the time) could suggest that this lack of group differences might be due to a floor effect. However, a similar floor effect should thus be expected also in the EZM test, conducted following exposure to adult stress. This was not the case; J + A rats were found to be significantly less active during the test (as presented in 2.2). Thus, the EPM results seem to reflect the relative lack of effect of the exposure to juvenile stress alone on exploratory behavior.

### 2.2. The Exposure to Juvenile Stress Affected Performance in the Two-Way Shuttle Avoidance Task in Adulthood 

**Figure 2:**
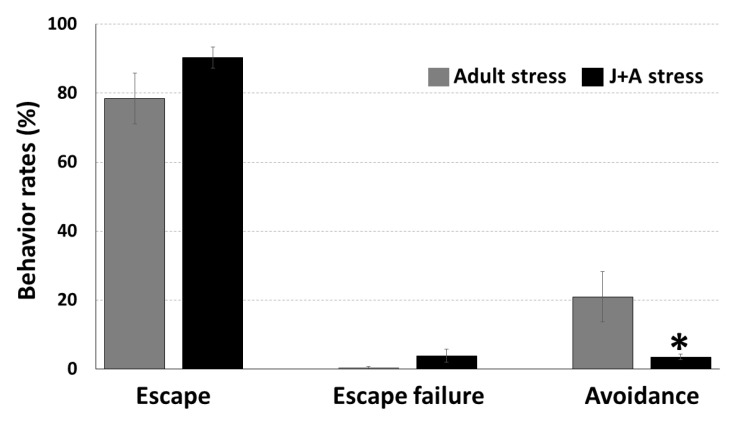
Rat behavior in the two-way shuttle avoidance task: Rats that were previously exposed to juvenile stress (J+A stress; *n* = 14) showed significantly lower rates of avoidance responses during the Two-Way Shuttle Avoidance (TWSA) test (the adult stress procedure) compared to rats that were exposed to the adult stress procedure without a prior exposure to juvenile stress (i.e., adult; *n* = 14). * Significant difference from adults; *p* < 0.05).

Two-way shuttle avoidance test was conducted 31 days after exposure to juvenile stress. While no significant differences were found between juvenile stress exposed and adult control rats in rates of escape (t(26) = 1.47, n.s.) and escape failure (t(26) = 1.78, *p* = 0.096) behaviors, a significant impairment of avoidance behavior was found in the juvenile stress exposed animals (Figure 2) (t(26) = 2.37, *p* = 0.034). Adult control rats’ rate of avoidance was 20.9% ± 7.3, while that of juvenile stress-exposed animals was only 3.5% ± 0.8. It is important to note that the significant difference in avoidance responses was not translated into a marked difference in the amount of foot shocks the animals experienced. This is because most animals (including juvenile stress exposed animals) very quickly exhibited a very efficient escape response (as can be seen in the levels of escape responses in Figure 2). Thus, they escaped to the other compartment almost immediately after the presentation of the tone, as was reported before [9,10].

### 2.3. The Effects of Combined Juvenile Stress and Stress in Adulthood

**Figure 3:**
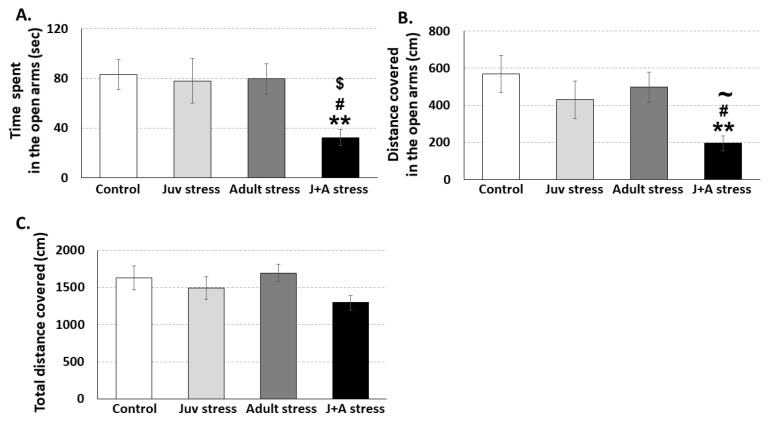
Rat behavior in the elevated zero maze (EZM) following exposure to the adulthood stress with or without preexposure to juvenile stress: J + A rats (*n* = 14) spent less time in the open quadrants of the EZM compared to all other groups (**A**). J + A rats showed lower activity levels in the open quadrants of the EZM compared to control (*n* = 14) and adult stress-exposed rats (*n* = 14) and a trend towards reduced activity compared to juv stress exposed rats (*n* = 14) (**B**). No significant reduction in total activity levels in the EZM was observed (**C**). ** Significant difference from control, *p* < 0.01; $ significant difference from juv stress, *p* < 0.05; # significant difference from adult stress, *p* < 0.05; and ~ border line significant difference from juv stress, *p* < 0.06.

The TWSA task had a dual purpose; it was used to examine the impact of juvenile stress on the ability to learn under stress in adulthood (Figure 2) and as an additional exposure to stress in adulthood. Thirty-four days after the exposure to juvenile stress and 3 days after the exposure to the adult stress (TWSA), animals with or without previous history of exposure to the juvenile stress were tested for anxiety-related behaviors in the Elevated zero maze (EZM). Overall, Multivariate analysis of variance (MANOVA) revealed a significant effect for the interaction between juvenile and adult stress exposures on rats behavior in the EZM (F(9,121) = 2.3, *p* = 0.044). Specifically, a significant effect for the interaction was found on the amount of time spent (F(3,55) = 3.5, *p* = 0.022( and activity levels (F(3,55) = 3.7, *p* = 0.017) in the open quadrants of the EZM. No significant main effect was found for the interaction between juvenile and adult stress exposures on total activity levels in the EZM area (F (3,55) = 1.7, n.s) (Figure 3). Further post hoc comparisons using the *Fisher’s Least Significant Difference* LSD test revealed that J + A rats spent less time in the open quadrants of the EZM compared to control (*p* = 0.007), juvenile stress exposed (juv stress; *p* = 0.015), or adult stress exposed rats (adult stress; *p* = 0.012) (Figure 3A). Post hoc comparisons also revealed that J+A rats covered less distance in the open quadrants of the EZM compared to control (*p* = 0.003), showed a borderline significant reduction compared to juvenile stress exposed (juv stress; *p* = 0.053), and showed a significant reduction compared to adult stress exposed rats (adult stress; *p* = 0.014) (Figure 3B).

We further examined whether the combined juvenile and adult stress exposure affected exploration behavior and the natural tendency to explore more an unfamiliar animal. Total exploration levels were calculated by measuring the total amount of time the rat spent exploring the familiar and the unfamiliar animal (in seconds). The social interaction index scores were calculated as the time spent exploring the familiar animal divided by the total time spent exploring.

**Figure 4:**
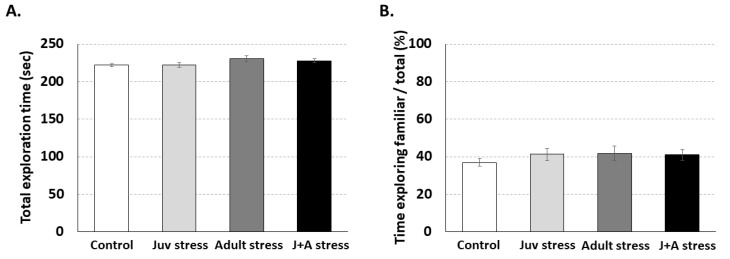
Social interaction test: No significant differences between the groups were found in total exploration time (**A**) and the amount of time spent exploring the familiar animal versus the unfamiliar animal (**B**).

Multivariate analysis of variance (MANOVA) did not revealed a significant effect for the interaction between juvenile and adult stress exposures on rats behavior in the social interaction test (F(6,102) = 0.2, *p* = n.s). Specifically, no significant differences were found between the groups in total exploration time (F (3,55) = 0.99, n.s.; Figure 4A). in addition, animals from all groups exhibited the expected tendency, and no significant differences were found between groups (F (3,55) = 0.15, n.s.; Figure 4B).

### 2.4. Expression of GABAAR and KOR

**Figure 5:**
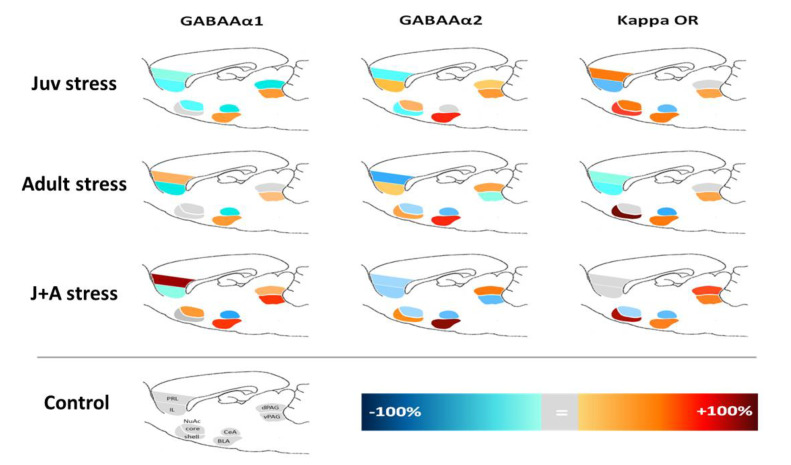
A combined map of γ-Aminobutyric acid type A (GABAA) and κ-opioid receptors (KOR) expression in a fear memory-related network of regions: Warm colors represent a positive shift from control group values, while cold colors represent a negative shift. The gray color represents no differences from the control. PRL, prelimbic; IL, infralimbic; NuAc, nucleus accumbens; CeA, central amygdala; BLA, basolateral amygdala; PAG, periaqueductal gray. (Control: *n* = 13; juv stress: *n* = 11; adult stress: *n* = 13; and J + A stress: *n* = 14.)

A complex pattern of stress-related alterations in the expression of GABAAR α1 and α2 subunits and KOR following juvenile stress, adulthood stress, or their combination was found, which was not easy to comprehend. Numerical data of these alterations is given in Appendix A. Overall, Multivariate analysis of variance (MANOVA) revealed a significant effect for the interaction between juvenile and adult stress exposures on expression patterns of GABAAR α1 and α2 subunits and KOR (F(3,47) = 3.17, *p* = 0.003). Specifically, following a *Bonferroni correction for multiple comparisons* significant effect of juvenile and adult stress, an interaction on expression pattern was found for GABAAR α2 in vPAG (F(1,47) = 5.35, *p* = 0.003). To help visualize the network impact of the stressors, we have constructed a combined expression image presenting the relative difference in expression of the different receptors. This image can emphasize the construct of the network and its gradual change between the different stress conditions (Figure 5).

To compare the expression patterns of GABAAR and KOR in the network of emotion regulation related regions following the combined exposure to juvenile and adult stress, we conducted multiple multinomial logistic regressions. At the first stage, we conducted an analysis for the expression of each receptor in the combined network of regions and used it to predict group classification. Classification successes and significant effects of the different region/receptor expression alterations are given in Appendix B. At the second stage, we have integrated those variables with the most significant contributions found in the first stage, combining different regions and receptors together in order to predict group classification using a wide expression network analysis. Expression levels were normalized to control group values that were used as reference in all analyses. The combined analysis was found highly significant (χ^2^(30) = 113, *p* < 0.001) with classification successes of over 90% (Table 1).

While group classification was satisfying, calculated odds ratios using the normalized expression values for each animal across every region and receptor type revealed no significant prominent contribution for a specific region or receptor type (Table 2), emphasizing the added value of a network level analysis.

## 3. Discussion

We used the juvenile stress paradigm in order to examine the combined effect of stress in juvenility and in adulthood on pathologic behaviors and the expression of GABAAR and KOR in the mPFC, NAc, amygdala, and PAG. Our results indicate that exposure to juvenile stress alone did not significantly affect anxious-like behaviors in adulthood. However, the exposure of animals to juvenile stress affected their ability to cope with additional stress in adulthood. Thus, altering their response from the adaptive avoidance response to escape and escape failure responses in the TWSA was found previously [50]. Moreover, augmentation in anxiety-like behavior in the EZM was found mainly in rats that were exposed to stress both in juvenility and adulthood compared to animals that were exposed only to the adult stress. These results are in line with others which have observed altered behaviors in adulthood following previous exposure to juvenile stress [9,10]. These findings largely resemble data of human populations [51]. Although previous studies have demonstrated the ability of early life stress to impair social behavior [52], our study SI test score did not differ between the groups. This may suggest a difference between the effects of early life stress (in the first two weeks of the pups) and of stress in juvenility [53]. The results anyway suggest that the impact of juvenile stress may affect more anxious-like behaviors.

Even long after the exposure to stress, the susceptible individual may inappropriately react to imminent stress and further develop PTSD [54]. Our findings accentuate the relevance of the juvenile stress model in the study of developmental psychopathology.

The current study points to the importance of the shift from micro- to macro-perspectives of stress-related neuromodulation. Stress responses require the orchestrated reaction of several brain regions together. Thus, compatible analyses of expression levels under the conception of network neuromodulation and their relation to the behavioral outcome of the stress are required. In line with that, when tested in each of the regions separately, the combined effect of juvenile stress and adult stress exposures had only minor effects on GABAAR and KOR expression levels (though few significant differences, which may be of importance, were found in group means; Appendix A). Assessment of the multidimensional involvement of the different receptor expressions in a network comprised of higher and lower emotion processing regions, such as the mPFC and PAG, and their known interactions with other limbic system structures [32,55] resulted in a highly successful classification. Several neurotransmitters are involved in the transmission of signals to and from the PAG. Specifically, GABAergic receptors are involved in descending pain control pathways, mediated by interneurons [56]. It was shown that stimulation of 5-HT on GABAergic neurons in the vPAG demonstrates anxiolysis [57]. Thus, receptors within the PAG can become a specific focus for pain research. As such, the evidence that presynaptic GABA receptors are more sensitive highlights alpha-2 receptor agents in pain control under certain conditions [58]. We suggest that a better description of the wide neuromodulation changes which occur in parallel in several brain regions (i.e., network activity) better captures the effect of stress and its functional significance. Furthermore, the accumulated predictive power of our classification analysis further increased when KOR expression was added to GABAAR subunit-altered expression, emphasizing the importance of integrating both regional and biochemical dimensions into a complex but more relevant characterization map.

The involvement of the GABAergic system [41] and the opioid system [48] in psychopathological outcomes of trauma exposure was proposed separately before. Our findings indicate that the combined neuromodulation changes of these receptors’ expressions cooccur in parallel and are part of the network effect of the exposure to the stress. Applying a network analysis to more comprehensively map trauma-related functional alterations enables a better understanding of those changes and their relevance to pathological manifestations.

## 4. Materials and Methods

### 4.1. Animals

Fifty-six male Sprague–Dawley rats aged 22 days (35–49 g; Harlan, Jerusalem, Israel) were used in the study. Animals were housed 2 per cage, in 35 × 60 × 18 cm Plexiglas cages in temperature-controlled (23 °C ± 1 °C) animal quarters on a 12:12-h. light–dark cycle (lights on 07–19 h.) in the local vivarium. Animals had ad libitum free access to standard rodent chow pellets and water.

### 4.2. Study Design

**Figure 6:**
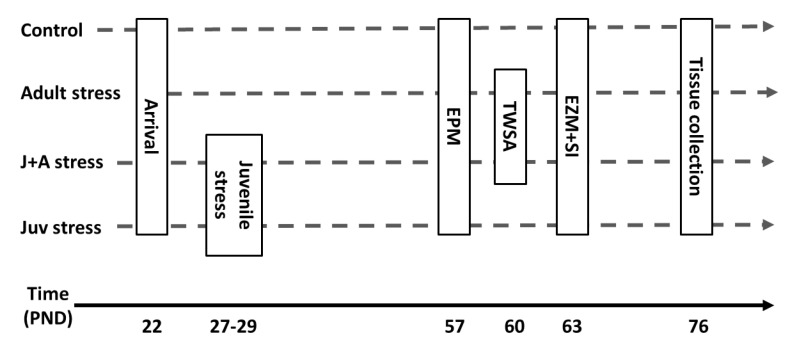
Experimental design.

As depicted in Figure 6, rats were randomly assigned to one of four groups:

(1) Juvenile stress group (“juv stress”): animals exposed to “juvenile stress” at juvenility (27–29 PND) and to the EPM, EZM, and SI tests in adulthood (57 + PND).
(2) Juvenile stress + adult stress group (“J + A stress”): animals exposed to “juvenile stress” at juvenility (27–29 PND); to the TWSA as “adult stress”; and to the EPM, EZM, and SI tests in adulthood (57 + PND).
(3) Adult stress (adult stress): animals exposed to the TWSA as “adult stress” and to the EPM, EZM, and SI tests in adulthood (57 PND).
(4) Control group (control): animals not exposed to any stress procedure but only to the EPM, EZM, and SI tests in adulthood (57 PND).

### 4.3. Behavioral Procedures

#### 4.3.1. “Juvenile Stress”

The juvenile stress procedure comprised of three consecutive days during which rats were exposed to different acute stressors on each day (adapted from [11]):

PND 27—forced swim—rats were placed in a plastic tank (diameter 50 cm and height 60 cm) containing water (22 ± 2 °C) 30 cm deep for 10 min.

PND 28—elevated platform—rats were placed for three sessions of 30 min each on a small, 12 cm × 12 cm elevated platform 70 cm above floor level. The intersession interval was 30 min during which animals were returned to their home cages.

PND 29—restraint—rats were placed for 2 h in a metal restraining box (11 cm × 5 cm × 4 cm) that prevented forward–backward movement and limited side-to-side mobility but did not discomfort the animal in any other way.

#### 4.3.2. Elevated Plus Maze Test

The EPM consists of two open arms and two closed arms (with 35 cm high walls) arranged in a way that similar arms are opposite to each other and elevated to 70 cm above the ground level. Following 5 min of habituation to the room, the animal was placed in the center of the maze, facing an open arm, and was allowed to explore the arena for 5 min. During this time, rat’s behavior was tracked and recorded by the Etho-Vision system (Noldus Information Technology, Wageningen, Netherlands). Time spent in open arms, distance traveled in the closed arms, open arms, and total were analyzed.

#### 4.3.3. “Adult Stress”—Two Way Shuttle Avoidance

These assessments were performed in the TWSA apparatus (Panlab harvard apparatus, Barcelona, Spain), as described before [9]. Rats went through one session of 75 “trace conditioning” trials. Conditioned Stimulus (CS): maximum of tone for 10 sec; unconditioned Stimulus (US): immediately following the CS termination an electric shock (0.7 mA) delivered for a maximum of 10 sec; and ITI: randomly varying 30 sec ± 20%.

Rats could produce one of three responses:

Avoidance—shuttling to the adjacent compartment upon hearing the CS. Following shuttling to the adjacent compartment, the tone was stopped and an ITI commenced; the rat avoided the electric shock.

Escape—shuttling to the adjacent compartment while the shock was on. The shock stopped, and an ITI commenced; the rat only reduced the duration that it was exposed to the shock.

Escape failure—failing to move to the adjacent compartment. The ITI commenced at the completion of the 10 s electric shock. The rat was subjected to the full duration of the electric shock.

#### 4.3.4. Elevated Zero Maze Test

The EZM was originally designed as a modification of the EPM. It comprises an elevated annular platform (90 cm diameter and 10 cm width) elevated to 70 cm above the ground level. It had two opposite, enclosed quadrants (with walls 35 cm height) and two open quadrants. Following 5 min of habituation to the room, the animal was placed in one of the open quadrants, facing a closed part of the apparatus, and was allowed to explore the arena for 5 min. During this time, rat’s behavior was tracked and recorded by the Etho-Vision system (Noldus Information Technology, Wageningen, Netherlands). Time spent in open quadrants, distance traveled in the enclosed quadrants, open quadrants, and total were analyzed.

#### 4.3.5. Social Interaction Test

The social interaction SCI test was conducted in a dimly-lit black Plexiglas arena (90 × 90 × 45 cm); two small metal grid compartments (20 × 20 × 35cm) were positioned in the center of the arena. One animal from the tested rat home cage was positioned in one compartment and served as the “familiar” rat, while another naïve animal was positioned in the other compartment and served as the “unfamiliar” rat. Following a 3 min habituation period to the testing room, rats were placed at the center of the arena for 5 min of free exploration. During this time, rat’s behavior was tracked and recorded by the Etho-Vision system (Noldus Information Technology, Wageningen, Netherlands). Time spent exploring the familiar animal divided by the total time spent exploring was analyzed.

### 4.4. Brain Tissue Harvesting

In previous studies published by our group, tissues were collected 24 h following the exposure to the adult stress [41]. This was done in order to assess the immediate effects of adult stress on receptors expression. In contrast, in the current study, the rationale was to assess the long-term organizational changes in receptor’s expression. Furthermore, in addition to reporting alterations in receptors’ expression, here, we suggest using the long-term biochemical modifications as a novel method for phenotyping the rats. Specifically, two weeks following the SI test, rats were decapitated; their brains were removed and quick-frozen using dry ice powder. Medial PFC (PRL and IL), NAc (core and shell), amygdala (CeA and BLA), and PAG (dorsal and ventral) regions were incised bilaterally with a sterile 1 mm punch at −20 °C using LEICA cryostat and according to the atlas of Paxinos and Watson (2007) [59]. The tissues were collected into 1.5 mL Eppendorf tubes and stored at –80 °C until further use. 

### 4.5. Biochemical Methods

#### 4.5.1. Homogenization

Tissues where homogenized in a glass Teflon homogenizer in 150 µl of ice-cold Urea Lysis Buffer ((1 mM EDTA (Fluka), 0.5% Triton X), 6 M Urea, 100 µM PMSF ((Sigma-Aldrich, St. Louis, MO, USA))) with freshly added protease inhibitor coctyle (complete—ROCHE) and phosphatase inhibitor (Dyn Diagnostic, Caesarea, Israel).

#### 4.5.2. Western Blot Analysis

Aliquots in SDS sample buffer were subjected to SDS–PAGE (10% polyacrylamide) and immunoblot analysis. Following 1 h semi-dry transfer onto a 0.45 µm nitrocellulose membrane, the lanes were compared for gross protein homogeneity loading by Ponceau staining (SIGMA). Blots were blocked using 5% Bovine Serum Albumin (BSA) in Tris-Buffered Saline Tween-20 (TBST: 0.9% *w/v* NaCl, 0.05% *v/v* Tween-20, and 100 mM Tris-HCl, pH = 7.6) incubation for 45 min at room temperature (RT). The membranes were incubated overnight on a shaker with first antibody in 3% BSA in TBST at 4 °C. The next day, excess of the first antibody was washed 3 times for 10 min with TBST. Secondary α-rabbit antibody incubation was conducted for 1 h at RT. The membranes were washed 3 times, 10 min each, in TBST before development, with EZ-ECL chemiluminescence light reaction (Amersham, Piscataway, NJ) using the CCD camera (XRS BioRad, Rishon Le Zion, Israel).

##### Reagents

Antibodies: GABAAR a1 subunit (cat. no. 224,203 (1:1000) rabbit polyclonal; SYSY), GABAAR a2 subunit (cat no. 224,103 (1:1000) rabbit polyclonal; SYSY), κ Opioid Receptor (KOR) (cat.no. 150,113 (1:1000) rabbit polyclonal; Biotest), β-Actin (N-19) (cat. no. sc-1616 (1:1500) polyclonal goat antibody; Santa Cruz Biotechnology, Santa Cruz, CA, USA), rabbit anti-goat (IgG) horseradish peroxidase (HRP) conjugated (Santa Cruz Biotechnology), goat anti-rabbit (IgG) horseradish peroxidase (HRP) conjugated, and the enhanced chemiluminescense (ECL+) kit were obtained from Amersham. All other chemicals were of analytical grade or the highest grade available.

##### Quantification

Quantification was performed using a CCD camera (XRS; Bio-Rad). The expression level for each depicted protein was calculated as the ratio between the band intensity of the protein divided by that of the normalized β-actin, a cytoskeletal protein used as an internal control. No differences were detected in β-actin levels throughout between the different groups.

### 4.6. Statistics

Results are expressed as means ± S.E.M. Behavioral indices were assessed by independent *t* tests, univariate or multivariate analysis of variance with post-hoc LSD tests where needed, and a sequential Bonferroni correction to correct for multiple comparisons in order to avoid type 1 errors [60]. To evaluate neuromodulation changes in the different regions in a comprehensive manner, the predictive power of GABAAR and KOR expression was assessed by multinomial logistic regression analyses, which is a predictive analysis.

### 4.7. Ethical Approval

The experiments were approved by the institutional Animal Care and Use Committee of the University of Haifa, and adequate measures were taken to minimize pain or discomfort, in accordance with the guidelines laid down by the NIH (Bethesda, MD, USA) regarding the care and use of animals for experimental procedures.

## Figures and Tables

**Table 1:** Results of the multinomial logistic regression for group classification.

Observed	Predicted				
	Control	Juvenile	Adult	Juvenile + Adult	% Correct
**Control**	**13**	0	0	0	100%
**Juv stress**	0	**13**	1	0	92.9%
**Adult stress**	0	1	**10**	2	76.9%
**J+A stress**	0	0	1	**13**	92.9%
Overall %	24.1%	25.9%	22.2%	27.8%	**90.7%**
χ^2^_(30)_ = 113, *p* < 0.001					

**Table 2:** Odds ratio for multinomial logistic regression predictors.

Explanatory Variables		Juv Stress		Adult Stress		J+A Stress	
Region+ Receptor	**Sig.**	**Odds Ratio**	95% CI	**Odds Ratio**	95% CI	**Odds Ratio**	**95% C**
**NuAc core GABAAα1**	0.000	**1.047**	0.824–1.331	**1.121**	0.873–1.438	**1.158**	0.896–1.496
**dPAG GABAAα1**	0.000	**0.874**	0.780–0.978	**0.970**	0.885–1.063	**1.020**	0.956–1.087
**vPAG GABAAα1**	0.052	**1.042**	0.884–1.228	**1.103**	0.927–1.312	**1.104**	0.929–1.314
**PRL GABAAα2**	0.005	**0.897**	0.715–1.124	**0.840**	0.663–1.065	**0.875**	0.695–1.102
**NuAc shell GABAAα2**	0.049	**0.987**	0.877–1.112	**1.056**	0.953–1.170	**1.043**	0.941–1.155
**BLA GABAAα2**	0.000	**1.146**	0.909–1.445	**1.176**	0.929–1.488	**1.187**	0.936–1.504
**vPAG GABAAα2**	0.000	**1.240**	0.894–1.720	**1.073**	0.775–1.486	**1.011**	0.734–1.394
**IL KOR**	0.190	**0.976**	0.896–1.063	**0.971**	0.887–1.062	**1.000**	0.908–1.101
**BLA KOR**	0.071	**1.160**	0.867–1.552	**1.149**	0.862–1.532	**1.140**	0.854–1.523
**CeA KOR**	0.063	**0.808**	0.530–1.231	**0.793**	0.515–1.219	**0.779**	0.502–1.210

**Figure A1:**
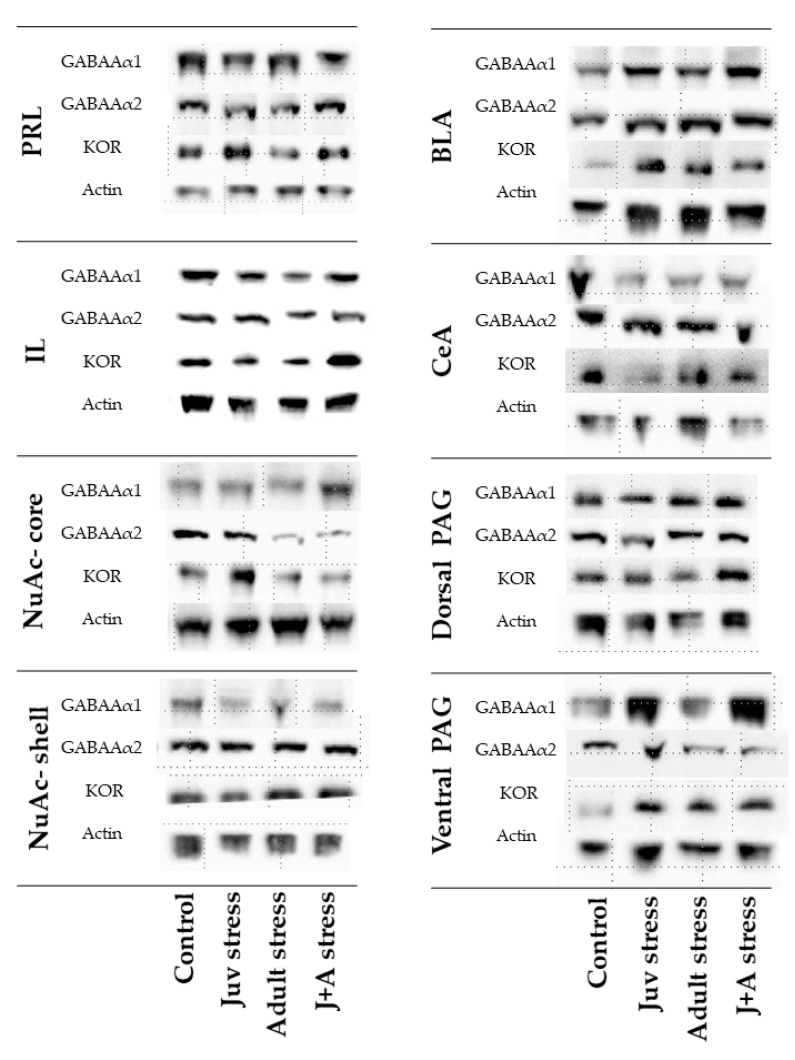
Representative blots of stress-related alterations in expression of GABAAR α1 and α2 subunits and KOR following juvenile stress, adulthood stress, or their combination: Sample blots are line blots from the same gel.

## Abbreviations

PTSD	Posttraumatic stress disorder
GABA	Directory of open access journals
mPFC	Medial prefrontal cortex
NAc	Nucleus accumbens
PAG	Periaqueductal gray
BLA	Basolateral amygdala
TWSA	Two-way shuttle avoidance task
EZM	Elevated zero maze
EPM	Elevated plus maze

## Appendix A

**Table A1:** Means ± S.E.M. for specific receptors expression and two-way ANOVA outcomes with LSD post hoc test (*p* < 0.05).

Region and Receptor Type	Control	Juv Stress	Adult Stress	J+A Stress	F Value	Sig.
**PRL**						
GABAAα1 GABAAα2 KOR	100 ± 10.7 100 ± 10.5 100 ± 8.3	87 ± 7.8 81 ± 7.9 145 ± 29.5	131 ± 30.0 60 ± 8.1 89 ± 11.0	219 ± 67.4 83 ± 14.5 108 ± 14.9	2.47 2.93 2.08	0.072 0.042 0.115
**IL**						
GABAAα1 GABAAα2 KOR	100 ± 9.4 100 ± 6.9 100 ± 11.1	80 ± 8.8 117 ± 17.8 71 ± 16.7	76 ± 15.8 108 ± 18.8 76 ± 25.4	88 ± 17.9 76 ± 13.0 95 ± 31.9	0.45 1.41 0.21	0.717 0.251 0.888
**NuAc-core**						
GABAAα1 GABAAα2 KOR	100 ± 7.3 100 ± 6.6 100 ± 9.8	79 ± 8.1 128 ± 18.7 146 ± 32.6	104 ± 16.4 82 ± 17.7 95 ± 12.4	139 ± 21.8 80 ± 12.6 86 ± 11.0	3.23 2.40 1.97	0.030 0.078 0.130
**NuAc-shell**						
GABAAα1 GABAAα2 KOR	100 ± 11.4 100 ± 13.2 100 ± 25.2	94 ± 14.3 79 ± 8.1 159 ± 42.9	100 ± 27.5 122 ± 26.5 285 ± 105	100 ± 12.9 120 ± 21.2 200 ± 29.4	0.04 1.14 1.32	0.990 0.343 0.279
**BLA**						
GABAAα1 GABAAα2 KOR	100 ± 6.8 100 ± 9.1 100 ± 9.7	138 ± 14.2 181 ± 40.0 134 ± 13.1	143 ± 22.6 186 ± 29.2 148 ± 15.5	156 ± 27.8 195 ± 21.9 139 ± 22.7	2.05 2.45 1.72	0.119 0.074 0.174
**CeA**						
GABAAα1 GABAAα2 KOR	100 ± 8.9 100 ± 9.2 100 ± 8.0	76 ± 12.4 93 ± 12.2 61 ± 7.8	75 ± 8.4 71 ± 16.4 56 ± 8.4	54 ± 10.4 70 ± 13.6 59 ± 12.9	1.82 1.35 2.98	0.156 0.268 0.040
**Dorsal PAG**						
GABAAα1 GABAAα2 KOR	100 ± 10.1 100 ± 7.4 100 ± 21.2	75 ± 8.5 112 ± 8.6 97 ± 10.2	106 ± 17.7 133 ± 20.1 101 ± 14.6	131 ± 36.4 147 ± 43.1 168 ± 32.5	1.13 0.56 2.32	0.346 0.640 0.086
**Ventral PAG**						
GABAAα1 GABAAα2 KOR	100 ± 11.9 100 ± 11.4 100 ± 10.4	140 ± 14.9 135 ± 16.2 132 ± 10.0	121 ± 19.6 90 ± 10.4 134 ± 14.2	165 ± 39.7 69 ± 9.2 *# 149 ± 24.7	1.34 5.35 1.32	0.270 **0.003** 0.279

* Significantly different from the control; # significantly different from juvenile are bold.

## Appendix B

**Table A2:** Classification successes and significant effects of multinomial logistic regression analyses conducted by separate receptors type.

**Classification by GABAAR α1.**					
**Observed**	**Predicted**				
	**Control**	**Juvenile**	**Adult**	**Juvenile + Adult**	**% Correct**
**Control**	**11**	1	1	0	84.6%
**Juv stress**	1	**10**	1	2	71.4%
**Adult stress**	3	3	**6**	1	46.2%
**J+A stress**	0	1	3	**10**	71.4%
Overall %	27.8%	27.8%	20.4%	24.1%	**68.5%**
χ^2^_(24)_ = 55.5, *p* < 0.001					
**Classification by GABAAR α2.**					
**Observed**	**Predicted**				
	**Control**	**Juvenile**	**Adult**	**Juvenile + Adult**	**% Correct**
**Control**	**10**	2	0	1	76.9%
**Juv stress**	3	**11**	0	0	78.6%
**Adult stress**	0	1	**9**	3	69.2%
**J+A stress**	1	1	3	**9**	64.3%
Overall %	25.9%	27.8%	22.2%	24.1%	**72.2%**
χ^2^_(24)_ = 77.1, *p* < 0.001					
**Classification by KOR.**					
**Observed**	**Predicted**				
	**Control**	**Juvenile**	**Adult**	**Juvenile + Adult**	**% Correct**
**Control**	**10**	0	1	2	76.9%
**Juv stress**	1	**6**	3	1	54.5%
**Adult stress**	1	2	**7**	3	53.8%
**J+A stress**	2	2	1	**9**	64.3%
Overall %	27.5%	19.6%	23.5%	29.4%	**62.7%**
χ^2^_(24)_ = 55.3, *p* < 0.001					

**Table A3:** Effects of receptors type in the separate analyses.

Brain Region	GABAAR α1 Sig.	GABAAR α2 Sig.	KOR Sig.
**PRL**	0.130	**0.006**	0.051
**IL**	0.051	0.051	**0.011**
**NuAc core**	**0.025**	0.029	0.254
**NuAc shell**	0.770	**0.000**	0.029
**CeA**	0.244	0.029	**0.001**
**BLA**	0.146	**0.000**	**0.013**
**dPA**	**0.006**	0.477	0.044
**vPAG**	**0.013**	**0.000**	0.105

Significance of regions chosen for further analysis is marked in bold.
